# Hepatitis C Virus: Evading the Intracellular Innate Immunity

**DOI:** 10.3390/jcm9030790

**Published:** 2020-03-13

**Authors:** Ana Rita Ferreira, Bruno Ramos, Alexandre Nunes, Daniela Ribeiro

**Affiliations:** Institute of Biomedicine—iBiMED, Department of Medical Sciences, University of Aveiro, 3810-193 Aveiro, Portugal

**Keywords:** hepatitis C virus, intracellular innate immunity, immune evasion, antiviral response, antiviral signaling

## Abstract

Hepatitis C virus (HCV) infections constitute a major public health problem and are the main cause of chronic hepatitis and liver disease worldwide. The existing drugs, while effective, are expensive and associated with undesirable secondary effects. There is, hence, an urgent need to develop novel therapeutics, as well as an effective vaccine to prevent HCV infection. Understanding the interplay between HCV and the host cells will certainly contribute to better comprehend disease progression and may unravel possible new cellular targets for the development of novel antiviral therapeutics. Here, we review and discuss the interplay between HCV and the host cell innate immunity. We focus on the different cellular pathways that respond to, and counteract, HCV infection and highlight the evasion strategies developed by the virus to escape this intracellular response.

## 1. Introduction

Hepatitis C virus (HCV) is still one of the most worldwide prevalent infectious agents, being responsible for an increasing level of liver-related morbidity and mortality and representing a global public health burden. Although the current therapeutic strategies can treat more than 95% of the diagnosed HCV-infected patients, the access to medical care and treatment is scarce and there is no efficient vaccine to prevent new infections [1,2,3]. Moreover, acute infection is typically asymptomatic and 70% of the patients may develop a chronic infection which can lead to chronic hepatitis, liver fibrosis, cirrhosis, and may even evolve to hepatocellular carcinoma [3].

HCV belongs to the *Hepacivirus* genus from the Flaviviridae family and is sub-divided into eight genotypes that differ according to geographic distribution, specific symptoms, and treatment response [1,4,5]. Until recently, the most common treatment method was based on the combined therapy of pegylated interferon-α (Peg-IFN-α) and ribavirin. Peg-IFN-α induces a cellular antiviral state and ribavirin acts in a synergistic manner through different mechanisms. Two of these mechanisms include the modulation of interferon-stimulated genes (ISGs) expression and error catastrophe induction by mutagen incorporation [6]. However, this therapy often resulted in adverse side effects. Nowadays, the most common treatment involves the use of direct-acting antiviral agents (DAAs). DAAs are divided into three main classes depending on their target proteins and genotype, and are combined to achieve optimal viral clearance (reviewed in [7]). The first developed DAAs consisted of protease inhibitors that targeted the NS3-4A complex. These were often combined with interferons (IFNs), and their efficacy was higher in patients infected with HCV-genotype 1. However, currently, a new generation of DAAs that target this protein are available and cover a wider range of genotypes. Although NS5A does not have enzymatic activity, it is the target of a class of DAAs, with a broad-spectrum genotype coverage but low efficacy in cases of viral resistance. Additionally, some DAAs target NS5B and are further divided in two subgroups: nucleoside analogues and non-nucleoside analogues. Nucleoside analogues are highly potent, have pan-genotypic activity and a high barrier against viral resistance. Non-nucleoside analogues are less efficient against a shorter number of HCV genotypes. Although the available treatments have shown to be quite effective, elimination of this disease may not be accomplished through treatment alone. However, the development of an effective vaccine remains a challenge due to the high genetic variability of the virus [1,8].

Upon infection of the host cells, viruses immediately trigger the intracellular innate immune defense signaling, expressing different pattern recognition receptors (PRRs) that recognize essential compounds of the viral structure, named pathogen-associated molecular patterns (PAMPs) [9]. PRRs can be present at the cytosol, bounded to cellular membranes, or secreted into the blood stream or into tissue fluids [10,11]. Intracellular PRRs recognize viral PAMPs that mainly consist of viral nucleic acids with specific signatures, allowing the differentiation between self and non-self [10,12]. PAMPs recognition activates different signaling cascades that ultimately induce the production of IFNs and ISGs, interfering with multiple steps of the viral infection.

Understanding the strategies employed by HCV to counteract the cellular innate immunity is of undeniable value to improve the current therapies and decrease liver disease, as well as to develop novel antiviral therapeutics. Here, we review the interplay between HCV and the host intracellular innate immunity, highlighting the evasion mechanisms employed by the virus to escape this cellular response. We furthermore discuss their implications to the outcome of infection and pathogenesis.

## 2. Hepatitis C Virus

**Figure 1:**
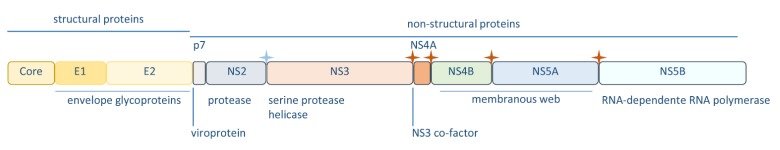
Schematic representation of the hepatitis C virus (HCV) polyprotein referring to the individual proteins and their relevance for the HCV life cycle. The stars indicate cleavage sites of auto-proteases—blue star: NS2-3; red star: NS3-4A. N-terminal processing is accomplished by host proteases.

HCV is an hepatotropic enveloped positive single-stranded RNA (+ssRNA) virus that primarily infects hepatocytes of humans and chimpanzees. However, the virus also has the capacity to spread to other cells, such as B cells and dendritic cells [13,14]. It has a 9.6 kb genome composed by an open reading frame that encodes a polyprotein containing 3000 amino acids, flanked by 5’ and 3’ untranslated regions (UTRs) at both ends (Figure 1).

**Figure 2:**
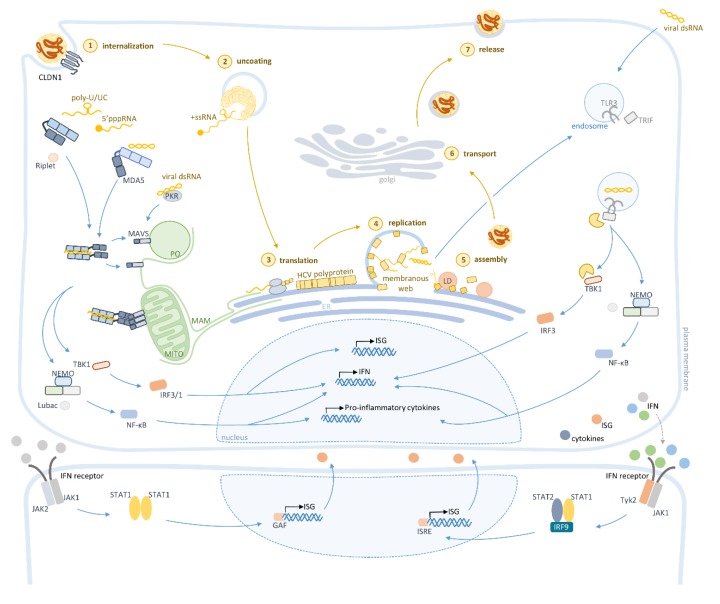
Hepatitis C virus (HCV) sensing by the intracellular innate immunity throughout the virus life cycle. Upon attachment to cellular receptors, HCV is internalized by endocytosis (1). Uncoating is then triggered and the viral genome is released into the cytoplasm (2). At the endoplasmic reticulum (ER) surface, viral RNA is translated (3), producing a polyprotein that it is co- and post-translationally processed into 10 viral proteins. Viral proteins’ expression induces the formation of a membranous web, which is essential for viral replication (4). HCV hijacks the lipid transport machinery for assembly (5) and uses the endosomal secretory pathway to be transported to the plasma membrane (6), where virion release occurs (7). The cytosolic sensors retinoic acid-inducible gene-I (RIG-I), melanoma differentiation-associated protein 5 (MDA5) and protein kinase R (PKR) together with the membrane pattern recognition receptor toll-like receptor 3 (TLR3) recognize viral RNA inducing the downstream activation of interferons (IFNs) and proinflammatory cytokines’ production.3. Activation of the Intracellular Innate Immunity by HCV.

NS4B expression induces alterations in intracellular membranes leading to the formation of ‘sponge-like inclusions’ closely associated with the rough endoplasmic reticulum (ER), designated as membranous web [15,16] (Figure 2), where genome replication takes place. NS5B is the RNA-dependent RNA polymerase (RdRp) necessary for the synthesis of a complementary negative-stranded RNA intermediate, which serves as a template for the amplification of the viral positive-stranded RNA genome. The HCV RdRp NS5B does not have proofreading activity leading to a high frequency of nucleotide substitutions during replications [17]. Consequently, there is a high error or mutation rate that leads to the rapid generation of viral variants. Thus, infected individuals present heterogeneous viral microvariants of the predominant master sequence, referred to as *quasispecies* [17,18]. The existence of such *quasispecies* is associated with different biological properties and phenotype in the host, such as carcinogenicity or tissue tropism. Moreover, *quasispecies* can led to different treatment outcomes: a correlation between *quasispecies* and resistance to IFN and DAAs treatment was recently reported [19].

HCV viral particle assembly is closely related to the cellular lipid metabolism [20]. After post-translation modifications, the core protein is transported to lipid droplets (LDs), inducing its relocation to a subcellular locus near the nucleus. LDs then become closer to the ER and to the membranous webs, where viral genome replication takes place [21], allowing the interaction between the core protein and the viral genome [22], and stimulating nucleocapsid formation [23]. The current models for virus particle envelopment suggest that nucleocapsids enter the ER lumen, where they obtain their lipid envelope, as well as the viral glycoproteins, through incorporation into the very low density lipoprotein (VLDL) pathway [20]. HCV virion release occurs through the endosome secretory pathway, which is regulated by the endosomal sorting complex required for transport (ESCRT), without inducing cell lysis [24,25].

## 3. Activation of the Intracellular Innate Immunity by HCV

HCV infection is mainly sensed by the cytosolic PRRs belonging to the family retinoic acid-inducible gene-I (RIG-I)-like receptors (RLRs) [26], DExD/H-box helicases that recognize different species of viral RNA [27]. The viral RNA is also recognized by the endosome membrane receptor toll-like receptor 3 (TLR3) [28,29], which belongs to the family of type I transmembrane receptors that recognize a diverse range of PAMPs (depicted in Figure 2) [30].

Upon HCV infection, RIG-I is quickly activated by the recognition of 5’ppp-RNA and poly-U/UC ribonucleotides in the 3’UTR region of the HCV genome (Figure 2) [26,31]. The contribution of MDA5 to HCV RNA sensing is still controversial. While Cao et al. have shown a greater contribution of MDA5 to the RLR signaling when compared to RIG-I, Hiet et al. demonstrated that MDA5 is only activated by double-stranded RNA (dsRNA) replication products produced during infection [32,33]. Nevertheless, RIG-I and MDA5 bind to the viral RNA backbone [32,33] and activate the mitochondrial antiviral signaling protein (MAVS) [34,35,36,37,38,39]. This interaction occurs via their CARD domains and induces a conformational change on MAVS, leading to the formation of resistant prion fiber-like active aggregates. These oligomers are essential to amplify the activation signal to other MAVS that are not directly activated [40,41]. MAVS polymerization recruits tumor necrosis factor (TNF) receptor-associated factors (TRAFs) (TRAF2, TRAF5, and TRAF6), which are required for the serine/threonine-protein kinase (TBK1) and the IκB kinase (IKK) complex activation [38,42]. These kinases are then responsible for the phosphorylation of the IFN-regulatory factor 3 (IRF3), as well as the nuclear factor-kappa-B (NF-κB). IRF3 dimerizes and translocates to the nucleus, where it prompts the expression of IFNs, cytokines, and ISGs [36,37,38,39].

TLR3 is activated later in HCV infection through the recognition of dsRNA intermediates that accumulate during HCV replication (Figure 2) [28,29]. TLR3 activates the toll/interleukin-1 receptor-domain-containing adapter-inducing interferon-β (TRIF) [43], which activates the transcription factors IRF3 and NF-κB, consequently inducing both the production of IFNs and inflammatory cytokines [12,43].

RIG-I and TLR3 signaling pathways converge into secreting IFNs to induce, in an autocrine and paracrine manner, the janus kinase/signal transducer and activator of transcription (JAK/STAT) pathway leading to the expression of ISGs, such as RIG-I, protein kinase RNA-activated protein (PKR), 2’,5’-oligoadenylate synthase (OAS), major histocompatibility complex (MHC) class I, and several others [44,45,46].

In the canonical pathway, IFNs bind to their respective surface receptors leading to the phosphorylation of specific residues at the associated JAK, which are consequently able to activate specific STAT residues, resulting in their nuclear translocation. Type II IFNs bind to their specific receptors inducing the homo-dimerization of STAT1, designated as gamma interferon-activated factor (GAF), which stimulates the expression of ISGs that have a gamma interferon-activated site (GAS). Type I and type III IFNs induce the hetero-dimerization of STAT1 with STAT2, which together with IRF9 form the IFN-stimulated gene factor 3 (ISGF3) to promote the expression of ISGs that contain interferon-stimulated response elements (ISREs). Recently, a far more complex, non-canonical signaling regulation of JAK/STAT pathway has been described [47,48].

## 4. Innate Immune Evasion Mediated by HCV

Antiviral defenses against HCV infection are rapidly established upon infection, even before extensive viral synthesis [31]. During acute HCV infection, and even in cases of high initial viremia, HCV can be spontaneously cleared, highlighting the importance of a rapid response by PRRs and innate immune induction [49]. However, 70% of HCV-infected patients do not effectively control the virus and develop chronic infection [3], suggesting that this virus is able to efficiently impair the host antiviral defenses.

**Figure 3:**
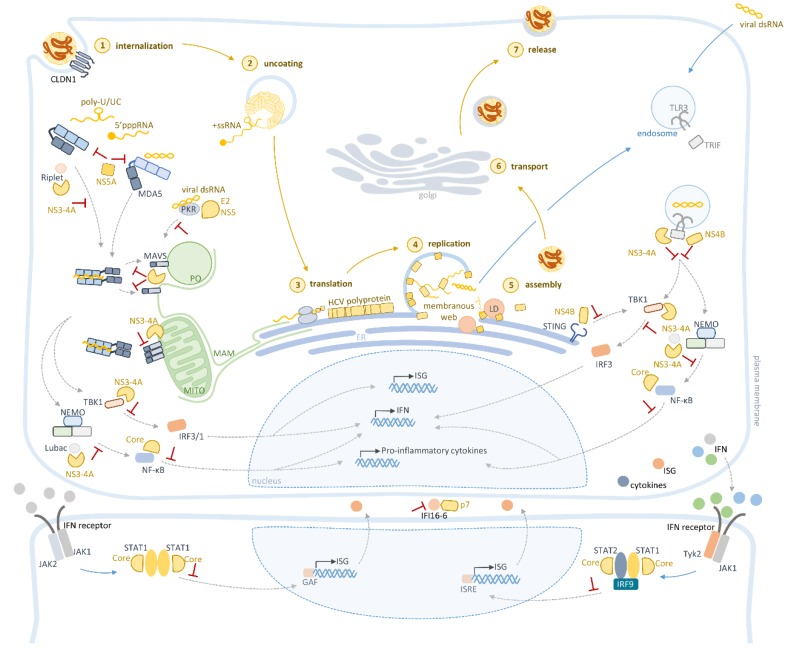
Evasion of the intracellular innate immunity response by hepatitis C virus (HCV) proteins. HCV NS3-4A, E2, NS5A, NS4B, p7, and core target several steps of the pattern recognition receptors (PRRs) signaling involved in HCV sensing, downregulating the expression of interferons (IFNs), proinflammatory cytokines and IFN-stimulated genes (ISGs), and consequently inhibiting the intracellular antiviral response.

HCV expresses a small number of proteins, which have multifunctional roles during infection. Besides being essential for viral replication, core, E2, NS3-4A, and NS5A also play important roles on immune evasion (summarized in Table 1 and depicted in Figure 3).

### 4.1. NS3-4A

The HCV NS3-4A protease is a complex formed by the serine protease NS3 and NS4A, a transmembrane domain that anchors NS3 to cellular membranes and allows the dimerization of the complex (Figure 1) [50]. Besides playing an important role in viral replication and assembly, NS3-4A is also essential for the cellular antiviral immunity evasion (reviewed in [50,51]).

NS3-4A is mostly recognized for cleaving MAVS at the membranes of mitochondria, peroxisomes, and mitochondrial-associated membranes (MAM) [31,37,52,53,54,55,56]. Cleavage by NS3-4A releases MAVS cytoplasmic domain, impairing the downstream signaling transduction, and consequent IFNs and ISGs expression [54,55]. This was even observed in the liver of HCV-infected patients, where patients that presented cleaved MAVS showed reduced levels of IFNs expression [31,57].

The HCV NS3-4A protease also cleaves TIR domain-containing adaptor protein-inducing IFN-β (TRIF), the adaptor protein of TLR3, suppressing IFN expression, as well as that of ISGs [28,58]. Due to HCV infection mainly being sensed by RLRs and TLR3 (Figure 2), the cleavage of these protein adaptors is critical for inhibiting the initial antiviral response set by the host.

By targeting both pathways, NS3-4A may also prevent excessive inflammation induced by HCV and/or block chemokine induction, delaying immune cell-mediated defense.

Besides targeting these critical adaptors of the two main antiviral sensing pathways, HCV NS3-4A also inhibits other components of the cellular innate immune response. Upon HCV activation of RLRs or TLR3, the corresponding signaling pathways converge to activate IRF3, a transcription promoter of IFNs by activating the kinase TBK1 [59,60]. It was reported that the NS3 helicase domain binds to TBK1, inhibiting its interaction with IRF3 and suppressing its translocation to the nucleus and consequentially impairing IFNs expression induction [61].

NS3-4A also targets Riplet, an E3 ubiquitin ligase, essential for RIG-I activation. It was suggested that NS3-4A cleaves Riplet, leading to the reduction of its endogenous levels and the consequential inhibition of RIG-I polyubiquitination [62]. More recently, Vazquez et al. reported that the NS4A Y16F residue is responsible for Riplet inhibition but does not impair MAVS cleavage. They further discovered that inhibition of IRF3 activation and IFNs production, through the impairment of Riplet activity, was independent of the RIG-I/MAVS signaling pathway [63].

Additionally, several reports have shown that HCV is able to inhibit NF-κB activation mediated by TNF-α, although the precise mechanism has never been elucidated [64,65]. Chen et al. showed that HCV NS3 binds to the linear ubiquitin chain assembly complex (LUBAC), responsible for the distinct polyubiquitylation of the NF-κB essential modulator (NEMO), which is required for the activation of NF-κB. This results in the inhibition of the expression of several inflammatory cytokines [66], and aids HCV persistence, since it may contribute to the limited inflammatory and immune responses.

Additionally, NS3-4A was also described to promote STAT1 degradation, although no direct interaction has been detected [67].

### 4.2. NS4B

NS4B has been shown to inhibit TLR3-mediated signaling by downregulating TRIF protein level, in a caspase 8-dependent manner [68].

Although HCV is an RNA virus, it has been shown that NS4B interacts with the stimulator of IFN genes (STING), impairing its downstream signaling [69,70]. STING is an adaptor protein localized at the ER membranes and essential for the induction of type I IFNs in response to viral DNA [71,72,73,74,75]. While it is unknown whether the STING pathway is activated by HCV, or even if it contributes to the antiviral response during HCV infection, it has been shown that activation of the STING pathway restricts viral replication in HCV-infected cells. [76]. The same study also showed differences on the effect of the STING antiviral response depending on the HCV genotype. Moreover, they report that the ability of NS4B to inactivate STING differs between HCV genotypes [76]. Although still controversial, the crosstalk between the RIG-I/MAVS RNA-sensing and STING DNA-sensing pathways, as well as the possible involvement of both signaling mechanisms in antiviral immunity against different RNA and DNA viruses, has already been suggested [70,77,78,79,80,81]. Whether this is the case for HCV infection remains to be elucidated but the current results seem to point in this direction.

### 4.3. NS5A and E2

HCV also targets PKR, an initiation factor 2a (eIF2a) phosphorylating kinase that is also a sensor for dsRNA, regulating IFN-β and ISGs production through MAVS, IRFs, and NF-κB [82,83,84,85]. However, regulation of PKR by HCV is a complex process since PKR can have both anti- and pro-viral functions. It was reported that PKR induces host translation shut-off, impairing the expression of antiviral effectors (pro-viral), and contributing for HCV infection persistence [84,86,87]. On the other hand, it was observed that PKR inhibits the expression of host factors, important for HCV replication, cell growth and survival, and induces IFN-β and ISGs production [88,89,90]. Nevertheless, it has been shown that HCV evades the PKR-mediated defense through NS5A and E2 [88,89,90]. It has also been demonstrated that HCV is able to induce PKR phosphorylation, activating its kinase function and inhibiting antiviral effector translation [84,86,87]. Recently, NS5A was also reported to interact with the nucleosome assembly protein 1-like 1 (NAP1L1), a nuclear-cytoplasmic chaperone reported to be involved in the regulation of several host pathways, such as transcription or cell cycle progression [91]. In HCV-genotype 2 infection, NS5A-NAP1L1 interaction results in the sequestration of NAP1L1, inducing its proteasomal degradation and impeding its nuclear translocation. NAP1L1 targeting downregulates the transcription of several genes essential for innate immunity, such as RIG-I- and TLR3-mediated responses [91]. Moreover, it has been shown that NS5A downregulates type I and type III IFNs activation induced by RIG-I- and MDA5-mediated responses. The authors suggested that NS5A binding to viral RNA may shield it from RIG-I and MDA-5 recognition [33].

### 4.4. Core 

The HCV core protein participates in multiple steps of the virus life cycle. Additionally, the core protein has been associated with alterations in several host biological functions, such as cellular growth and apoptosis [92,93,94]. The overexpression of this protein led to malignant transformation of rat fibroblasts and to hepatocellular carcinoma in transgenic mice [95,96,97].

The role of the HCV core protein in immune evasion is controversial, mainly regarding its function on the NF-κB pathway. Shrivastava et al. described that core protein overexpression blocked the NF-κB pathway upon stimulation with different NF-κB agonists [98]. Later, gene expression analysis of HepG2 cells expressing the core protein suggested that the NF-κB pathway was inhibited by this viral protein in order to suppress inflammatory responses [99]. However, several studies reported that the HCV core protein activates the NF-κB pathway [100,101,102,103] to induce an inflammatory response that characterizes the pathogenesis of HCV infection. These differences may be due to the different experimental setups and cells used in these studies, especially considering that experiments were mostly performed with transient transfection or inducible systems. Nonetheless, and taking into consideration the pathogenesis of HCV infection, it seems plausible that HCV infection would activate NF-κB to induce anti-apoptotic pathways and consequently the expression of proinflammatory cytokines.

While HCV has evolved several strategies to disrupt PRRs signaling and consequently impair the expression of type I IFNs, it has also developed specific mechanisms to counteract the JAK/STAT pathway required for cellular response to IFN-mediated stimulation. Several reports have shown that the core protein modulates this pathway, though there are some divergences regarding the mechanism involved. Initially, it was reported that the HCV core protein binds to STAT1 inducing its degradation [52,67,104]. It was also reported that the core protein inhibits the binding of STAT1 to DNA [105,106,107,108]. More recently, it has been suggested that the HCV core protein exerts different effects on IFN-α and IFN-γ, with the downregulation of the first and upregulation of the second [109,110,111]. Some studies show reduced levels of phosphorylation of STAT2 [110,111], similar to what was observed in HCV-infected patients [112]. Again, the observed discrepancies may be due to the distinct experimental setups used in these studies.

### 4.5. p7

A genome-wide mutagenesis study, performed to understand the anti-IFN functions of HCV, has shown that p7, a viral membrane-spanning protein that acts as an ion channel and is required for virion production [113], also has immune evasion functions. After measuring the impact of each liver-specific ISG on HCV replication, they found that p7-mutant HCV was susceptible to IFI16-16 overexpression, an ISG that is highly inducible upon type I IFN treatment of viral infections, but whose function remains to be elucidated. Qi et al. furthermore reported that p7 interacts with IFI16-16 and induces depolarization of mitochondrial membrane potential, which they suggest to inhibit IFI16-16 function [114].

Growing evidence demonstrates that HCV modifies host microRNA (miRNA) expression to modulate a diverse range of host functions [115]. It was reported that HCV induces the expression of miR-208b and miR-499a-5p, which impair the expression of IFNL3, a member of the IFN-λ family, allowing HCV to escape from innate immunity [116]. HCV was also reported to induce selenoprotein P (SeP), an hepatokine involved in insulin resistance and type 2 diabetes. Murai et al. showed that SeP mRNA binds and inhibits RIG-I activity, and that its overexpression is associated with poor treatment outcome. However, the mechanism behind SeP upregulation is not yet known. Interestingly, Grünvogel et al. have shown that a portion of HCV dsRNA intermediates are released from infected cells in extracellular vesicles, and further suggest that this mechanism contributes to the reduction of the activation of TLR3 [117].

## 5. Conclusions

The host cell innate immune system reacts to HCV infection by producing a variety of type I and/or type III IFN-induced ISGs. Nevertheless, this response only controls HCV infection to some extent, and is not sufficient to eliminate HCV from the large majority of the infected patients. This is mainly due to the fact that the virus has developed several mechanisms to counteract this antiviral response. These mechanisms involve multiple viral proteins and are specific to the different cellular antiviral signaling pathways that recognize and react against the virus. Together, these coordinated strategies build up an effective evasion from the host intracellular immune response. Here, we have described and discussed the currently recognized evasion strategies employed by HCV (summarized in Table 1 and depicted in Figure 3). However, more of these mechanisms will certainly be unraveled in the near future.

The available DAAs for treatment of HCV infection have revolutionized healthcare and constitute highly effective treatment options.

However, these drugs are expensive and unavailable for the majority of the patients. Furthermore, there is a genuine concern for drug resistance and unwanted secondary effects. There is, therefore, an urgent need to develop an effective vaccine to prevent HCV infection and to ultimately eliminate the disease. Additionally, it is essential to develop new therapies to decrease the morbidity and mortality of HCV-induced liver disease. Further studies on the interplay between HCV and the host intracellular immune signaling may prove to be valuable to better understand the progression from acute to chronic infection, as well as access the role of host innate immunity in hepatic inflammation. Moreover, these studies may unravel possible new cellular targets for the development of novel antiviral therapeutics.

## Figures and Tables

**Table 1:** Roles of hepatitis C virus (HCV) proteins on the evasion of the intracellular antiviral response.

Viral Factor	Function	Reference
N3-4A	cleaves MAVS, to impair production of IFNs and proinflammatory cytokines	[31,37,52,53,54,55,57]
	cleaves TRIF, to impair production of IFNs and proinflammatory cytokines	[28,58]
	inactivates Riplet, inhibiting RIG-I and IRF3 activation	[62,63]
	binds to LUBAC, impairing the polyubiquitynation of NEMO required for NF-κB activation	[66]
	induces degradation of STAT1, impairing the expression of antiviral effectors	[67]
	binds to TBK1, impairing IRF3 activation	[59,60,61]
Core	blocks NF-κB, to suppress inflammatory response	[98,99]
	targets JAK/STAT pathway by targeting STAT1 and STAT2, inhibiting the production of ISGs	[52,67,104,105,106,107,108,110,111,112]
E2	interacts with PKR, repressing its antiviral effects	[89]
NS5A	interacts with PKR, repressing its antiviral effects	[88,90]
	induces NAP1L1 degradation, inhibiting gene transcription essential for RIG-I- and TLR3-mediated responses	[91]
	impedes RIG-I- and MDA5 activation, impairing IFNs expression	[33]
NS4B	downregulates TRIF protein, inhibiting TLR3 signaling	[68]
	interacts with STING, inhibiting the production of IFNs	[69,70]
p7	interacts with IFI16-16, inhibiting its antiviral function	[114]
